# Employee equity incentive, executive psychological capital, and enterprise innovation

**DOI:** 10.3389/fpsyg.2023.1132550

**Published:** 2023-02-27

**Authors:** Liping Yu, Jiabin Hu

**Affiliations:** ^1^Business School, Changzhou University, Changzhou, China; ^2^School of Statistics and Mathematics, Zhejiang Gongshang University, Hangzhou, China

**Keywords:** employee equity incentive, senior executives’ psychological capital, enterprise innovation output, intermediary effect, regulatory effect

## Abstract

The input and deployment of human resources, such as senior executives and the core employees involved in different innovation processes, is key to developing enterprise innovation activities. Under the same framework, it is crucial to explore how employee equity incentive and senior executives’ psychological capital affect enterprise innovation. This paper’s research sample comprises listed companies that implemented equity incentives in the A-share market from 2010 to 2021, examining the relationship between executive psychological capital, and enterprise innovation. This study found that: (1) Employee equity incentive and executives’ psychological capital can significantly improve the quality of innovation output while promoting enterprises to increase the quantity of innovation outputs; (2) Executive psychological capital contributes to the intermediary effect between employee equity incentive and enterprise innovation; (3) R&D investment has a positive moderating effect on employee equity incentive, senior executives’ psychological capital and enterprise innovation; (4) The innovation incentive effect brought by the implementation of stock options by enterprises is more significant, which also makes the psychological capital of executives maintain a positive role in enterprise innovation; (5) In addition, the positive effects of employee equity incentive and executive psychological capital on enterprise innovation are affected by different property rights. The positive effects of employee equity incentive and executive psychological capital on enterprise innovation in state-owned enterprises are not high.

## 1. Introduction

The world’s economic structure will face subversive changes during centennial change. Presently, China’s economic form is already undergoing changes. As the micro-foundation of high-quality development, enterprises bear the goal of driving the improvement of national independent innovation capability. How to accelerate the number of innovation achievements and improve the quality of innovation output is the focus of academic circles. Unlike ordinary investment activities, enterprise innovation activities have high risks, large investment and a long cycle, which depend on the input of resources and the improvement of efficiency (Jefferson et al., 2006; Hirshleifer et al., 2012), among which the input and use of human resources are more critical (Cho et al., 2016). In the principal-agent chain of enterprises (shareholders – executives – employees), executives are in front of employees. The existing research mainly discusses how the psychological capital of executives (Gao et al., 2020; Wang et al., 2021) and the characteristics of senior executives (Chen et al., 2016) affect the innovation of enterprises by influencing the innovative behavior of employees. Employees are also an important part of the chain, and their efforts will directly affect the realization of the innovation goals of senior executives. In view of this, how to stimulate their willingness to innovate has become one of the hot topics. For enterprises, although executives determine the allocation of innovation input, the performance of enterprise innovation output is affected by core employees. The continuous innovation ability comes from the joint role of executives and core employees. It is extremely necessary to put employees and executives in the same framework to explore the relationship between them and their impact on enterprise innovation.

From the perspective of innovation process, senior executives dominate the investment of innovation resources. As a trait of senior executives, psychological capital has been proved to have a greater impact on innovation decision-making than other factors in many studies (Li et al., 2020). In the process of innovation, employees influence the effect of “R&D input into innovation output” transformation, employee equity incentives has the characteristics of long validity, wide range and prominent convexity, which makes it an indispensable compensation tool to motivate employees to innovate (Manso, 2011; Ederer and Manso, 2013). Currently, only a few literatures attempt to explore the internal mechanism of employee equity incentive affecting innovation. From the perspective of principal-agent relationship, some scholars believe that equity incentives can alleviate the senior executive–employee agency problem (Wu and Tu, 2007), improve employees’ sense of belonging and willingness to innovate (Mao and Weathers, 2019), and then improve the efficiency of enterprise innovation (Tsang et al., 2021), improve the sense of belonging among employees, thus improving enterprise innovation efficiency (Tsang et al., 2021). A few scholars also believe from the incentive theory that employee equity incentives have a classification effect, retaining talent with higher R&D enthusiasm and wind direction commitment (Hall and Murphy, 2003; Oyer and Schaefer, 2005). However, limited by people’s inherent cognition and data availability, the internal mechanism of core employee equity incentives that affect enterprise innovation still lacks empirical evidence, and most of the literature is still discussed and analyzed from the perspective of executives. From the above, it is necessary to answer what effect does employee equity incentives play on enterprise innovation, and how does senior executives’ psychological capital affect the relationship between the two?

This paper takes the listed companies with A-share equity incentive from 2010 to 2021 as the research sample. Explore the positive and negative effects of senior executives’ psychological capital and employee equity incentives on enterprise innovation, and the role of senior executives’ psychological capital in the latter two. Compared with previous studies, the innovative contribution of this research are: First, the existing psychological capital of senior executives is mostly measured by the scale questionnaire, which is difficult to put together with such variables from enterprise samples as employee equity incentives. The indicator system of psychological capital of senior executives developed in this paper can make it applied to more extensive empirical research. Second, the continuous innovation ability of enterprises comes from the joint action of senior executives and core employees. However, the existing literature rarely places the two under the same framework for research. This paper will expand this research in order to provide reference for the theory and practice of enterprise innovation management. Third, since enterprise innovation requires not only “incremental” but also “quality improvement” to understand the mechanism guiding employee equity incentives and senior executives’ psychological capital on enterprise innovation more comprehensively, this paper conducts research from the two levels of quantity and quality of enterprise innovation achievements. Considering that enterprise innovation needs not only “increment” but also “quality improvement,” in order to understand the mechanism of employee equity incentive and executive psychological capital on enterprise innovation more comprehensively, this paper conducts research from the two levels of quantity and quality of enterprise innovation achievements. Fourth, according to principal-agent theory，equity incentive effectively alleviates the agency problem between senior executives and employees, and promotes the interconnection among employees, senior executives and enterprises. From this perspective, it is worth examining the role played by senior executives’ psychological capital in employee equity incentives and enterprise innovation. Fifth, this paper provides empirical evidence of the difference in the effectiveness of executive psychological capital and employee equity incentives under varying property rights and forms of equity incentive.

## 2. Literature review

### 2.1. Literature on senior executives as the main body of enterprise innovation research

In the long run, the value advantage of enterprise innovation comes from the input and use of “people” (Baron and Armstrong, 2007). As innovation decision-makers, senior executives are critical for innovation. Limited by the characteristics of innovation, as well as the hedonism (Bernstein, 2015) and myopia (Fang et al., 2014) that executives may have, executives’ psychological capital plays an increasingly important role as a special enterprise resource capability in many studies on the effect of senior executives on enterprise innovation (Lafuente et al., 2019; Li et al., 2020). Executive psychological capital can be defined as the stable psychological characteristics and positive psychological state that senior executives possess. It is a “*state-like*” element that integrates personality traits and mental state, as well as a synthesis of, for example, self-efficacy and psychological resilience.

From the perspective of executives’ psychological capital, subsequent studies explored its impact on enterprise innovation from the following angles:

1. According to the agency theory, the psychological capital of leaders acts on innovation performance by exerting influence on themselves and employees. On the one hand, Managers with high-level psychological capital have stronger motivation to make plans, find resources to respond to innovation needs, and quickly rebound from innovation failures to conduct more innovative activities (Russo and Stoykova, 2015; Hallak et al., 2018). On the other hand, high psychological resilience will also produce a downward transmission effect, strengthen employees’ psychological resilience (Sihvola et al., 2022), improve employees’ innovation behavior (Wang et al., 2021).
2. How the heterogeneity of executives’ spirit of self-sacrifice, overconfidence, and experience affect enterprise innovation. With the deepening of research, a few scholars pointed out that senior executives’ personal characteristics, experiences, and even overconfidence would have a heterogeneous impact on innovation decisions (Mao and Zhang, 2018). Others agree that self-sacrificing leadership can greatly enhance employee cohesion, fulfill the regulatory role of psychological capital, and thus affect the effectiveness of team innovation (Van Knippenberg and Van Knippenberg, 2005).
3. The role of short-sighted constraints and risk-bearing capacity in enterprise innovation. The risk of innovation will aggravate the shortsightedness of senior executives (Fang et al., 2014), reduce their risk-bearing capacity and innovation willingness, which is not conducive to enterprise innovation.

### 2.2. Literature on the relationship between employees and enterprise innovation

**Figure 1:**
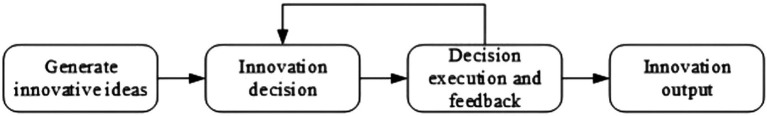
Enterprise innovation process.

At present, people have cognitive biases about the innovative effects of employees. With reference to the research of Zaltman et al. (1973) and other scholars, the innovation process can be divided into four stages (as shown in Figure 1): the formation of innovation ideas, innovation decisions (making innovation plans and resource allocation), decision implementation and feedback, and innovation output. Intuitively, executives have more influence on the innovation decision-making stage. Although employees have less influence and cannot participate in innovation decision-making, they play an irreplaceable role in the formation of innovation ideas (Bradley et al., 2016； Chang et al., 2015) and the implementation and feedback on decisions. The relationship between employee equity incentives and enterprise innovation has gradually aroused academic discussion in recent years. However, the relevant literature is still limited, and a consistent conclusion cannot be drawn.

From the perspective of principal-agent theory, in the principal-agent chain, the performance of employees affects the rights and interests of executives and shareholders, most studies believe that equity incentives can greatly alleviate employee–enterprise information asymmetry, connect employees and enterprises, enhance innovation cooperation and supervision among employees, and then improve innovation efficiency (Brander and Zhang, 2017; Tsang et al., 2021). From the perspective of incentive theory, some scholars pointed out that equity incentives can enhance employees’ psychological ownership perception and promote employees’ sense of belonging to the organization, while the classification effect can retain talent (Oyer and Schaefer, 2005; Torp and Nielsen, 2018), improve employees’ R&D enthusiasm (Mehta et al., 2017), and motivate employees to share technology and knowledge (Mao and Weathers, 2019). In addition, equity incentives enhance employees’ willingness to take risks. In addition, equity incentives enhance employees’ willingness to take risks.

### 2.3. Literature on the relationship between executives, employees, and enterprise innovation

Improvements to the innovation level depend not only on the decisions of senior executives but also on implementation by core employees. Therefore, the academic community began conducting enterprise innovation research based on both executives and employees to maximize their role in enterprise innovation. Initially, they applied their research perspective to the executive–employee pay gap and enterprise innovation (Ding et al., 2009). In this regard, there are two opposing views: First, based on championship theory, the widening pay gap can effectively stimulate innovation willingness among lower-level employees, reduce supervision costs, and may help improve innovation performance (Burns et al., 2013). Second, considering the higher risk of improving the quality of innovation and the existence of social comparison theory, the widening of the salary gap is not conducive to team cooperation and reduces employee innovation enthusiasm for high-quality innovation achievements, ultimately damaging the quality of innovation (Siegel and Hambrick, 2005). As equity incentives have become the main incentive mechanism used by enterprises, some studies have begun comparing the effects of such incentives on executives and employees to determine the main group responsible for incentive innovation. Some scholars pointed out that senior executives’ equity incentives cannot directly affect innovation efficiency by driving output through innovation input (Edmans et al., 2017), while employee equity incentives can increase innovation output by improving innovation efficiency (Hochberg and Lindsey, 2010). In addition, some scholars discussed the relationship between executives’ overseas experience, employees’ skills, and enterprise innovation (Hattori and Lapidus, 2004), while others considered building a model of executives, employees’ attention, and innovation output (Muller and Whiteman, 2015).

### 2.4. Review

From the above, it is evident that the areas worthy of further supplementary research include the following:

1. The effectiveness of core employees in enterprise innovation and how employee innovation enthusiasm could be stimulated.
2. The effect of employee equity incentive in the process of enterprise innovation: Limited by data availability, more scholars only discuss the theoretical mechanism that employee equity incentive affects enterprise innovation, but neglect to provide evidence for it.
3. Executive psychological capital may play a mediating role in the impact of employee equity incentives on enterprise innovation: Starting from agency theory, Equity incentive can effectively alleviate the agency problem between executives and employees, promote the connection between them and the enterprise, improve innovation efficiency, and in turn affect executives’ decision-making.
4. The impact of R&D investment on senior executives’ decision-making and the implementation effect of employees; the effect of employee equity according to the type of equity incentive, and; the impact of varying property rights and institutional background on the effectiveness of executive psychological capital and employee equity incentives: R&D investment is not only a key variable influencing senior executives’ decision-making, but also a guarantee in determining the implementation effect of employees, which may play a regulatory role. Furthermore, the effect of employee equity incentives should vary according to the type of equity incentive. In addition, when combined with China’s institutional background, the effectiveness of executive psychological capital and employee equity incentives may vary significantly under different property rights. However, existing research seems to afford little consideration to these three points.

## 3. Theoretical analysis and hypothesis presentation

### 3.1. Theoretical analysis of the impact of employee equity incentive on enterprise innovation

In the enterprise, senior executives and core employees are at an important link in the principal-agent chain. When combined with the four stages of the innovation process, we can see that innovation activities cannot be carried out without the innovation decisions made by senior executives and the efforts of core employees who implement the decisions. The efforts of key employees directly determine the effect of the transformation of “R&D input into innovation output.” In fact, the absence of the role of core employees, that is, the interests of shareholders, executives, and employees, and where employees are inconsistent, may not be conducive to the innovation output of enterprises. Employee equity incentives provide an effective solution to this problem. The research advances three reasons why equity incentives for core employees are conducive to accelerating enterprise innovation.

First, From the perspective of principal-agent theory, on the one hand, employee equity incentives alleviate the shareholder–employee agency problem, connecting their rights and interests, these incentives can effectively stimulate employees’ willingness to innovate, improve employees’ sense of belonging to the enterprise (Torp and Nielsen, 2018), and reduce the talent turnover rate. On the other hand, the implementation of employee equity incentives can encourages employees to supervise each other, forming a more lasting incentive effect. In addition, they can encourage senior executives to increase investment and improve innovation efficiency (Tsang et al., 2021).

Second, From the perspective of incentive theory, the classified incentive effect generated by equity incentives can facilitate the retention of talent with higher innovation enthusiasm and initiative, effectively guaranteeing the vitality of internal R&D in enterprises.

Third, long term effective equity incentive can avoid the emergence of shortsightedness of core employees, improve their willingness to bear risks, increase their tendency to favor long-term benefits, and induce improvements in innovation efficiency (Chang et al., 2015).

According to the above theoretical analysis, the stronger the equity incentive given to core employees, the better the incentive effect. Under the same innovation conditions, enterprises will get more and higher quality innovation output. Therefore, the following assumption can be made:

*H1a*: The higher the intensity of equity incentive given to employees, the greater the positive effect on the quantity of innovation output of enterprises.

*H1b*: The higher the intensity of equity incentives given to employees, the more conducive to improving the quality of enterprise innovation output.

### 3.2. Theoretical analysis of the impact of executives’ psychological capital on enterprise innovation

As a special enterprise resource capability, many studies have confirmed that the enhancement effect of senior executives’ psychological capital on innovation performance is far greater than that of other resource capabilities (Li et al., 2020). In this paper, senior executives’ psychological capital is defined as the stable psychological characteristics and positive psychological state of senior executives, which synthesizes self-efficacy, emotional stability, psychological resilience, and other dimensions. This paper advances the following reasons for the strong effect of senior executives’ psychological capital on enterprise innovation:

First, In the principal-agent chain of enterprises, senior executives are in front of employees, and the psychological capital of senior executives has a downward transmission effect, On the one hand, senior executives have a strong sense of self-efficacy, and their confidence can engender an environment conducive to organizational innovation. On the other hand, it can significantly affect employees’ innovation behavior (Wang et al., 2021), and enhance their psychological resilience, sense of organizational belonging, and even risk-bearing level, thus affecting innovation efficiency. Second, according to “time-oriented theory,” executives’ subjective preferences for the future greatly affect the innovation in and development of enterprises. Executives with higher psychological capital are more concerned about the long-term impact of the enterprise. They optimize their professional and strategic visions and tend to be optimistic about the future value of enterprise innovation, driving them to be more decisive regarding existing innovation decisions and effectively improving innovation performance. Third, the experience of senior executives, their personal characteristics, and other factors affect innovation decision-making (Hambrick and Mason, 1984). Long cycle, high investment and high risk innovation activities make senior executives unable to judge innovation performance, which may lead to short-sighted behavior. At this time, senior executives with high levels of psychological capital show resilience, making them more risk-tolerant (Fatoki, 2018) and more willing to innovate.

Based on the foregoing, corresponding assumptions can be made:

*H2a*: The psychological capital of executives has a positive impact on the quantity of innovation output of enterprises. The higher the psychological capital, the greater the positive effect.

*H2b*: The psychological capital of executives has a positive impact on the quality of innovation output of enterprises. The higher the psychological capital, the greater the positive effect.

### 3.3. Theoretical analysis of the relationship between employee equity incentive, executive psychological capital, and enterprise innovation

#### 3.3.1. The intermediary effect of senior executives’ psychological capital on employee equity incentive and enterprise innovation

The development of enterprise innovation activities requires movement through many links. The only parts that can be controlled by human resources are R&D resource input and enterprise innovation output. Among them, senior executives play a decisive role in the amount of innovation input. As direct innovators, the efforts of core employees greatly affect the transformation effect of “input into output” and the feedback on innovation decisions. The psychological capital and risk preference of senior executives are key factors in decision-making. In practice, it is found that relying solely on senior executives to discover investment opportunities and determine resource allocation may not improve innovation performance. Moreover, the shortsightedness of senior executives and the principal–agent problem involving employees make senior executives psychologically prefer short-term returns and low-risk innovation activities.

According to agency theory, employees are at an important link in the principal-agent chain, and their efforts directly affect the realization of the innovation goals of senior executives and the rights and interests of senior executives. Employee equity incentives can effectively alleviate the senior executive–employee agency problem, stimulate the innovation ability of employees, and bring better positive market reaction for enterprises (Fang et al., 2015). It makes senior executives pay more attention to realizing the enterprise’s long-term value, enhances their willingness to take risks, and gives them a stronger internal motivation to deal with high-risk investments; Starting from the innovation process and combining the incentive theory, equity incentive for core employees can produce innovation incentive effect, increasing their diligence and motivation to innovate, thereby effectively improving the input–output conversion rate and innovation benefits. Also, implementing these incentives plays a positive feedback role in innovation decisions, in turn, strengthening senior executives’ psychological capital and urging these executives to gradually increase R&D investment in high-risk, innovative projects.

From this perspective, employee equity incentives can positively impact enterprise innovation by enhancing senior executives’ psychological capital and improving their risk preferences. Accordingly, we advance the following assumption:

*H3a*: The psychological capital of executives plays an intermediary role between employee equity incentives and the quantity of enterprise innovation outputs.

*H3b*: The psychological capital of executives plays an intermediary role between employee equity incentives and the quality of enterprise innovation outputs.

#### 3.3.2. The impact of the forms of employee equity incentive on the effectiveness of employee equity incentives and executive psychological capital

Stock option and restricted stock are two forms of equity incentive for Chinese listed companies. The return and risk of equity option forms are not equal, which will constitute the incentive effect of sharing risks. The value of restricted stock positively correlates with the stock price of the enterprise so that the employees can obtain certain returns, which may produce the risk aversion effect. On the one hand, it may reduce the risk tolerance of core employees, thus leading to the enterprise’s lack of overall innovation motivation. On the other hand, because the rights and interests of senior executives are related to enterprise performance (Minnick and Noga, 2010), the risk aversion effect of employee equity incentives makes senior executives more inclined to low-risk, high-return projects, hindering high-quality innovation activities with greater risk.

Based on the foregoing, corresponding assumptions can be made:

*H4a*: Compared with the implementation of stock options, the implementation of restricted stock will reduce the positive effects of employee equity incentive and executive psychological capital on the quantity of innovation output.

*H4b*: Compared with the implementation of stock options, the implementation of restricted stock will reduce the positive effects of employee equity incentive and executive psychological capital on the quality of innovation output.

#### 3.3.3. The innovation effect of employee equity incentive and executives’ psychological capital is affected by the nature of property rights

When combined with China’s institutional background, under different property rights, Whether the effectiveness of employee equity incentives and senior executives’ psychological capital have a positive effect on enterprise innovation is affected by different property rights. State owned enterprises enjoy more policy support and subsidies than non-state-owned enterprises but also face many government goals, tasks, and policy constraints. As for the “executive executives” of state-owned enterprises, their political promotion motivation urges them to pay more attention to their “achievements” in office, leading to them having low enthusiasm for innovation and a low willingness to take risks. They may attach importance to innovation output in the short term and ignore encouraging enterprises to carry out high-quality innovation activities. For the core employees of state-owned enterprises, in an environment with many policy restrictions, plans regarding employee equity incentives issued by these state-owned enterprises tend to be welfare-based, limiting the incentive effect of the equity plan and making it difficult to mobilize employees’ motivation for innovation.

Based on the foregoing, corresponding assumptions can be made:

*H5*: The positive effects of employee equity incentive and executive psychological capital on enterprise innovation are affected by different property rights. The positive effects of employee equity incentive and executive psychological capital on the quantity and quality of innovation output of state-owned enterprises are not high.

**Figure 2:**
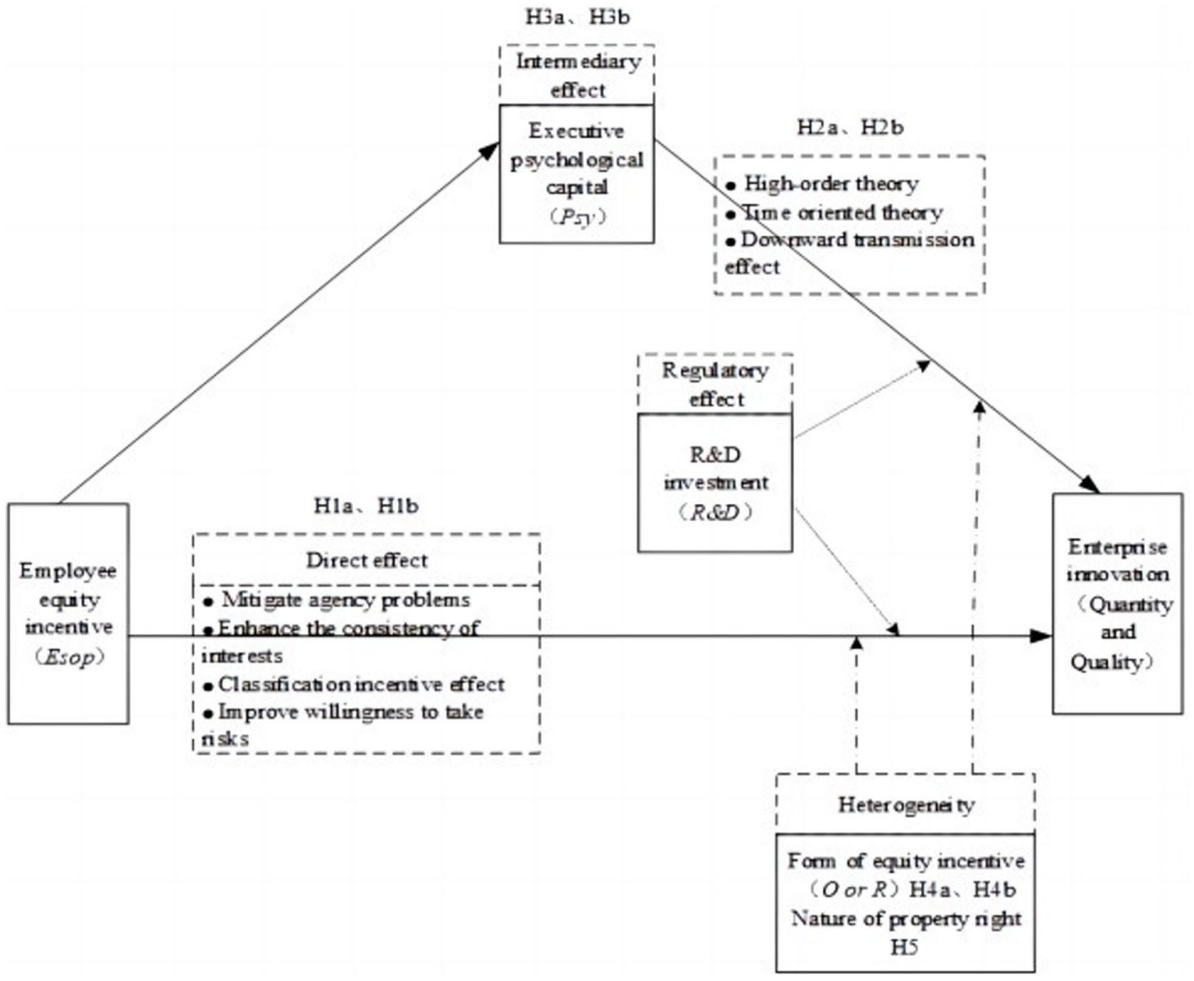
Theoretical model.

In summary, this paper’s theoretical model can be summarized as follows (Figure 2):

## 4. Research design

### 4.1. Sample selection and data source

This paper uses relevant data from the CSMAR database to select companies listed on the A-share market that implemented equity incentives from 2010 to 2021 as its research sample. On this basis, this study screens the data on senior executives’ psychological capital, innovation output, R&D input, and other relevant variables. The filter conditions are as follows:

1. It considers the particularity of financial enterprise data and the instability of ST and * ST company data. Both parts of the relevant data are eliminated.
2. Equity incentives for core employees is the key explanatory variable in this paper. The data of companies that do not carry out equity incentives for employees and only carry out equity incentives for senior executives are excluded.
3. Executive psychological capital is also a key explanatory variable applied in this paper, and related data is matched with employee equity incentives data.
4. In this paper, the number of patent and invention patent applications are used to measure the quantity and quality of enterprise innovation. In this regard, companies without patent applications or invention patent application data are excluded.

After manually screening relevant data according to the above conditions, 669 observations are obtained to assess the impact of executive psychological capital and employee equity incentive on the number of examples of enterprise innovation. Furthermore, 567 observations are obtained to study the impact of executive psychological capital and employee equity incentives on the quality of enterprise innovation. The data obtained is unbalanced panel data.

### 4.2. Variable selection and definition

#### 4.2.1. Explained variable: Innovation output (Innovation)

By referring to the research in relevant fields, these indicators are selected to measure the two levels of enterprise innovation output: quantity and quality. In this study, the innovation output at the enterprise quantity level will be represented by the total number of patent applications (including inventions, utility models, and design patents) (*T_Innovation*). Although the number of patents granted and the number of patent applications can reflect the innovation output of enterprises, compared with patent authorization, patent applications can more accurately depict real-time patent innovation (Griliches, 1990) and better reflect the innovation efficiency of enterprises. In addition, the innovation output of enterprise quality can be represented by the total number of invention patents (*Q_Innovation*). International scholars believe that patents of great importance and high technical levels will be cited more frequently. So, in their research, they often use the number of patent citations by enterprises in that year to measure the quality of innovation output (Bradley et al., 2016). However, patent citations take time, and there is a lack of relevant statistics in China. However, due to their required large investment in R&D, high technology content, and high-value achievements, invention patents can better demonstrate the substantive innovation levels of enterprises, and are an appropriate indicator of the quality of enterprises’ innovation output.

#### 4.2.2. Explanatory variable: Employee equity incentive intensity (*Esop*)

The Administrative Measures for Equity Incentives of Listed Companies stipulates that the objects of equity incentives can be senior executives, directors, core technicians, and core business personnel. In this paper, the latter two are defined as “core employees.” Regarding research in related fields (Call et al., 2017), the employee equity incentive intensity is represented by the proportion of incentive shares (options and restricted stocks) granted to core employees in equity incentive plans among the total number of shares of the company is selected. It should be noted that although the newly added or accumulated grants can be used for measurement, it is more logical that this paper selects the newly added grants for the core employees’ incentive shares, considering that employees may exercise their rights after reaching performance goals and being granted incentives.

#### 4.2.3. Mediating variable: Executive psychological capital (*Pyc*)

The psychological capital of senior executives has the nature of both personal emotions and personality traits, and its connotation is the positive psychological state and psychological characteristics of senior executives (Luthans et al., 2007; Russo and Stoykova, 2015). It includes three sub-dimensions: self-efficacy, emotional stability, and psychological resilience. In this paper, the entropy weight method is used to determine the weight of multi-dimensional indicators. Then, the indicators are synthesized to obtain personal psychological capital, while the psychological capital of the company executives included in the study sample is obtained by averaging the sum of all executives’ psychological capital.

##### 4.2.3.1. Self-efficiency

The sense of self-efficacy refers to the confidence of senior executives regarding their future expectations and their own strengths. It is generally believed that the more educated an individual, the higher their cognitive learning ability and knowledge reserve level. Therefore, they should be more willing to bear risks in innovation investment (Huang and Sheng, 2013) and show higher self-confidence. Moreover, the longer the senior executive serves, the stronger their management experience and professional competence, and the higher their self-efficacy when operating in a complex decision-making environment. Therefore, this paper selects senior executives’ educational background and tenure as indicators to measure self-efficacy. The educational background of senior executives is measured by the equivalent weighting method. The educational background of technical secondary school and lower is assigned a value of one, that of junior college is assigned a value of two, an undergraduate education background is assigned a value of three, that of a master’s degree is assigned a value of four, and a doctoral educational background doctor is assigned a value of five. The tenure of office is expressed according to the time over which the senior executives included in the sample companies have held their current positions.

##### 4.2.3.2. Emotional stability

Emotional stability reflects executives’ control over their emotions. Many scholars believe that as they grow older, the emotional threshold of senior executives will rise due to their rich experience, they will not be easily disturbed by the outside world, and their emotional stability will be high. Some scholars also pointed out that the fairness of salary will significantly affect the psychological balance and behavior choice of executives, and the fairness of relative salaries can effectively adjust the conflict between managers and shareholders. Therefore, this paper selects age and relative salary as indicators to measure emotional stability. Age is expressed by the current age of senior executives from sample companies. Relative compensation is expressed by comparing executive compensation with the average of all executive compensation given by the company in the same year.

##### 4.2.3.3. Psychological resilience

As an integral part of positive psychological capital (Rego et al., 2021), executive psychological capital refers to the ability to effectively cope with stress, adversity, frustration, and other situations, and quickly recover from psychological conditions (Den Hartigh and Hill, 2022). The psychological resilience of senior executives is affected by their personal experiences (Santoro et al., 2020). For example, senior executives with advanced academic backgrounds demonstrate more rigorous thinking, stronger self-discipline, and perseverance in exploration (Francis et al., 2015), and the intellectual capital they have enables them to deal with problems more calmly and rationally. As another example, senior executives with overseas backgrounds, including educational and professional experiences, have incurred more economic and energy costs, greatly improving their anti-pressure ability. Therefore, this paper selects academic background and overseas background as indicators to measure psychological resilience. Academic and overseas backgrounds are measured by the equal weight assignment method. There is no relevant background in Assignment 1; however, there is relevant background in Assignment 2 (see Table 1).

**Table 1:** Index system of senior executives’ psychological capital.

Variable	Level I indicators	Weight	Secondary indicators	Weight
*Pyc*	*Self-efficacy*	0.4034	*Degree*	0.2359
			*Duration*	0.1675
	*Emotional stability*	0.2651	*T-age*	0.0593
			*Relative*	0.2058
	*Psychological resilience*	0.3315	*Academic*	0.1988
			*Overseas*	0.1327

#### 4.2.4. Adjusting variable: R&D investment intensity (*R&D*)

Review the relevant literature (Aghion et al., 2013; Cornaggia et al., 2015). Existing studies mainly measured the intensity of R&D investment through “R&D investment/(total assets or market value or operating income).” The operating income of the sample of listed companies selected for this study is vulnerable to profit manipulation, operation management, and other factors, and the market value is difficult to estimate. Therefore, this paper measures the intensity of R&D investment by taking the “R&D expenses/total assets” in the annual reports of the sample companies.

#### 4.2.5. Control variables

Combing relevant literature for reference (Chang et al., 2015; Fang et al., 2015; Call et al., 2017). Combined with the actual development of Chinese enterprises, the following variables are selected as this study’s control variables: Enterprise scale (*Size*); Capital intensity (*Tangibility*); Enterprise age (*Age*); Enterprise performance (*ROA*); Lever level (*Lev*); Intangible assets (*Inlc*); Enterprise equity concentration (*Vrd*) (see Table 2).

**Table 2:** Variables definition.

Variable properties	Variable names	Symbol	Variable calculation
Interpreted variables	Quantity of innovation outputs	*T_ Innovation*	Ln(1 + Total number of patent applications)
	Quality of innovation output	*Q_ Innovation*	Ln(1 + Number of invention patent applications)
Explanatory variable	Staff equity incentive intensity	*Esop*	Total number of shares granted to key employees/Total number of company shares
Intermediary variable	Executive psychological capital	*Pyc*	Self-efficacy, Emotional stability, Psychological resilience (Entropy weight method)
Regulating variable	R&D investment intensity	*R&D*	R&D expenses/Total assets
Control variables	Enterprise scale	*Size*	Natural logarithm of total assets
	Capital intensity	*Tangibility*	Net fixed assets/Total assets
	Enterprise age	*Age*	Value year - Establishment year (natural logarithm)
	Enterprise performance	*ROA*	Net profit/Total assets
	Lever level	*Lev*	Total liabilities/Total assets
	Intangible assets	*Inlc*	Natural logarithm of net intangible assets
	Enterprise equity concentration	*Vrd*	Shareholding ratio of the largest shareholder (%)

### 4.3. Research model design

#### 4.3.1. Employee equity incentive, senior executives’ psychological capital, and enterprise innovation output

This paper mainly examines the direct relationship between employee equity incentive, executive psychological capital and enterprise innovation output, and examines the impact of executive psychological capital and employee equity incentive on enterprise innovation output in terms of quantity and quality by building Models (1) and (2):


(1) $$\begin{array}{l} T\_Innovation_{it}=c+\alpha Esop_{m,it}+\chi Pyc_{it}+\\ \underset{j}{\sum }\beta_{j}control_{it}+\lambda_{i}+\mu_{it} \end{array}$$

(2) $$\begin{array}{l} Q\_Innovation_{it}=c+\alpha Esop_{m,it}+\chi Pyc_{it}+\\ \underset{j}{\sum }\beta_{j}control_{it}+\lambda_{i}+\mu_{it} \end{array}$$

In Formulae (1) and (2), “*T_Innovationit*” and “*Q_Innovationit*” represent the quantity and quality of enterprise innovation output, respectively; “*Esop_it_*” refers to employee equity incentives, “*α*” is the elastic coefficient of these incentives, “*c*” is a constant term, “*control_it_*” is a series of control variables, and “*μ*” represents the random disturbance term.

This paper seeks to alleviate the problem of heteroscedasticity and sequence correlation that may occur in the regression of the two models, as well as improve the robustness of the regression results by making a robust adjustment to the standard error of the regression coefficient.

#### 4.3.2. The intermediary effect of senior executives’ psychological capital on employee equity incentive and enterprise innovation

This paper tests the intermediary effect executives’ psychological capital may play on employee equity incentive and enterprise innovation (i.e., to test hypothesis H3) by applying Baron and Kenny, 1986 intermediary test method for reference and analyzing it by combining the stepwise regression method with the Sobel test. First, from the level of quantity and quality of innovation output, the study builds the Recursive Models (3) and (4) as follows:


(3) $$\left\{\begin{array}{c} \begin{array}{l} T_{\_Innovation_{it}}=c_{1}+\gamma Esop_{m,it}+\\ \underset{j}{\sum }\beta_{j}control_{it}+\lambda_{i}+\mu_{it} \end{array}\\ \begin{array}{l} Pyc_{it}=c_{2}+\alpha ESop_{m,it}+\\ \underset{j}{\sum }\beta_{j}control_{it}+\lambda_{1}+\mu_{it} \end{array}\\ \begin{array}{l} T_{\_Innovation_{it}}=c_{3}+\gamma Esop_{m,it}+\delta Pyc_{m,it}+\\ \underset{j}{\sum }\beta_{j}control_{it}+\lambda_{i}+\mu_{it} \end{array} \end{array}\right.$$

(4) $$\left\{\begin{array}{c} \begin{array}{l} Q_{\_Innovation_{it}}=c_{1}+\gamma Esop_{m,it}+\\ \underset{j}{\sum }\beta_{j}control_{it}+\lambda_{i}+\mu_{it} \end{array}\\ \begin{array}{l} Pyc_{it}=c_{2}+\alpha ESop_{m,it}+\\ \underset{j}{\sum }\beta_{j}control_{it}+\lambda_{1}+\mu_{it} \end{array}\\ \begin{array}{l} Q_{\_Innovation_{it}}=c_{3}+\gamma^{\prime }Esop_{m,it}+\delta Pyc_{m,it}+\\ \underset{j}{\sum }\beta_{j}control_{it}+\lambda_{i}+\mu_{it} \end{array} \end{array}\right.$$

The specific analytical process for the intermediary effect is as follows:

1. Analyze the regression of employee equity incentives to enterprise innovation, using the regression coefficient *γ*. If it is not significant, there is no intermediary effect. If γ is significant, proceed to the next step.
2. Test the regression coefficient in turn *α*、and *γ*´. If both coefficients are significant, there is a mediating effect. If at least one is not significant, the Sobel test should be conducted, and an assessment should be made according to the significance of the test results.
3. Test the regression coefficient after obtaining the results of the intermediary effect according to the previous step on *δ*. If it is not significant, then senior executives’ psychological capital plays a complete intermediary effect. On the contrary, such an outcome reveals a partial intermediary effect.

#### 4.3.3. The moderating effect of R&D investment on employee equity incentive, senior executives’ psychological capital, and enterprise innovation

This paper tests the moderating effect of R&D investment on employee equity incentive, executives’ psychological capital, and enterprise innovation (i.e., to test hypotheses H4 and H5), by applying the hierarchical adjustment regression analysis method to assess the moderating effect. The following model can be constructed by taking the moderating effect of R&D investment on employee equity incentives and enterprise innovation as an example:


(5) $$\left\{\begin{array}{l} \begin{array}{l} T\_Innovation_{it}=c_{1}+\alpha_{1}Esop_{e}{{}_{,}}_{it}+\\ \underset{j}{\sum }\beta_{j}control_{it}+\lambda_{i}+\mu_{it} \end{array}\\ \begin{array}{l} T\_Innovation_{it}=c_{2}+\alpha_{2}Esop_{e}{{}_{,}}_{it}+\chi Esop_{e,it}\times R\&D_{it}+\\ \underset{j}{\sum }\beta_{j}control_{it}+\lambda_{i}+\mu_{it} \end{array} \end{array}\right.$$

(6) $$\left\{\begin{array}{l} \begin{array}{l} Q\_Innovation_{it}=c_{1}+\alpha_{1}Esop_{e}{{}_{,}}_{it}+\\ \underset{j}{\sum }\beta_{j}control_{it}+\lambda_{i}+\mu_{it} \end{array}\\ \begin{array}{l} Q\_Innovation_{it}=c_{2}+\alpha_{2}Esop_{e,it}+\chi Esop_{e,it}\times R\&D_{it}+\\ \underset{j}{\sum }\beta_{j}control_{it}+\lambda_{i}+\mu_{it} \end{array} \end{array}\right.$$

The model constructed by testing the moderating effect of R&D investment on executives’ psychological capital and enterprise innovation is similar to Model (6) above, so it will not be repeated. According to the above Models (5) and (6), the significance of regression coefficient χ after the cross-multiplication of R&D (regulating variable) and *Esop* can be tested and analyzed to determine whether R&D investment has a regulating effect.

### 4.4. Research model design

Table 3 presents the descriptive statistical results of this paper’s main variables from the two levels of the quantity and quality of enterprise innovation output. The innovation output at the quantitative level is measured by the total number of patent applications, while the output at the qualitative level is represented by the number of development patent applications. Table 3 shows that the average value of the number of innovation output is 3.772, the maximum value is 9.373, while the average value of the quality of innovation output is 2.916 and the maximum value is 8.951. These data show that existing enterprises still focus onthe quantity of the achievement output in innovation, whose overall quality level is not high. The average value of employee equity incentive is 0.023 and 0.022, respectively, indicating that the sample companies only have about 2% equity incentive for core employees, and the intensity of equity incentive is low. The standard deviation of senior executives’ psychological capital and employee equity incentive is small, indicating that the dispersion of relevant data is stable and less affected by abnormal and extreme values. In addition, the standard deviations of other control variables are within a reasonable range and are appropriately representative.

**Table 3:** Descriptive statistics of variables.

Variables	*T_Innovation*				*Q_Innovation*			
	Average value	Min	Standard deviation	Max	Average value	Min	Standard deviation	Max
*Innovation*	3.775	0.693	1.371	9.373	2.916	0.693	1.337	8.951
*Esop*	0.023	0.001	0.022	0.155	0.022	0.001	0.021	0.155
*Pyc*	7.154	5.453	0.508	9.345	7.150	5.984	0.519	9.345
*R&D*	7.721	0.110	9.724	167.410	7.989	0.020	10.455	167.410
*Size*	21.986	19.744	1.105	26.237	21.808	19.744	1.028	26.237
*Tangibility*	0.163	0.003	0.119	0.669	0.161	0.003	0.114	0.611
*Age*	2.705	0.693	0.382	3.526	2.688	1.386	0.384	3.526
*ROA*	0.063	−0.439	0.060	0.295	0.062	−0.439	0.056	0.295
*Lev*	0.355	0.034	0.177	0.891	0.335	0.034	0.170	0.789
*Inlc*	0.041	0.001	0.046	0.486	0.037	0.001	0.033	0.322
*Vrd*	32.508	4.080	14.164	81.180	31.381	4.080	13.757	75.170

## 5. Empirical results

### 5.1. Multicollinearity diagnosis of variables

The choice of multiple variables makes the regression analysis more comprehensive, but the multicollinearity problem among independent variables may also lead to the reduction of the accuracy of the parameter estimation during the regression, so that the influence degree of independent variables cannot be accurately judged. Based on this, it is necessary to carry out multicollinearity diagnosis for the selected variables before regression. The common way is to use SPSS software to carry out variance expansion factor test (VIF test). When the VIF is less than 10, it is proved that there is no strict collinearity problem between variables. According to the collinearity diagnosis results (Table 4), the VIF values of all variables in Model 1 and Model 2 are less than 3 and close to 1, which proves that there is no collinearity problem between variables, and the settings of Model 1 and Model 2 are more reasonable.

**Table 4:** Collinearity test result (VIF).

Variables	Model (1)	Model (2)
	*T_In*	*Q_In*
*Esop*	1.038	1.028
*Pyc*	1.111	1.092
*R&D*	1.297	1.359
*Size*	1.613	1.621
*Tangibility*	1.121	1.149
*Age*	1.137	1.139
*ROA*	1.356	1.318
*Lev*	2.290	2.229
*Inlc*	1.063	1.066
*Vrd*	1.062	1.047
*VIF mean*	1.309	1.305
*N*	669	567

### 5.2. Analysis of basic regression results

Table 5 mainly applies the model constructed above to conduct regression analyses on the relationship between senior executives’ psychological capital, employee equity incentives, and enterprise innovation. Model (1) reported the regression results of the impact of senior executives’ psychological capital and employee equity incentives on the number of enterprise innovation outputs. According to the results, the higher the intensity of employee equity incentives, the greater the positive impact on the number of enterprise innovation outputs. The positive impact coefficient is 0.175, which is significant at the 10% level. Similarly, there is also a significant positive correlation between senior executives’ psychological capital and the number of enterprises’ innovative outputs. The value of executives’ psychological capital influencing innovation is 0.152. Model (2) reported the regression coefficient value of the impact of senior executives’ psychological capital and employee equity incentive on the quality of enterprise innovation output. Based on the regression value, the impact coefficient of employee equity incentive was 0.105, which was significant at the 10% level, proving that the quality of the innovation output of enterprises that implement equity incentives for core employees will also be improved. In addition, the coefficient of executives’ psychological capital affecting the quality of innovation output is positive, 0.122. The improvement of innovation quality will inevitably cope with more uncertainty and higher risk. The positive psychological state of senior executives is an important resource capability for enterprise innovation. Synthesize the regression values in the following table, it can be seen that employee equity incentives and senior executives’ psychological capital have a significant positive effect on enterprise innovation output. Therefore, Hypotheses H1a, H1b, H2a, and H2b are tested.

**Table 5:** Senior executives’ psychological capital, employee equity incentive, and enterprise innovation.

Variables	Model (1)	Model (2)	IV-2SLS(1)	IV-2SLS(2)
	*T_Innovation*	*Q_Innovation*	*T_Innovation*	*Q_Innovation*
*Esop*	0.217^**^ (4.726)	0.105^*^ (2.278)	0.279^*^ (2.117)	0.148^*^ (1.700)
*Pyc*	0.163^*^ (2.564)	0.158^*^ (1.729)	0.144^*^ (1.758)	0.178^*^ (1.757)
*R&D*	0.208^***^ (4.074)	0.032^***^ (6.332)	0.217^***^ (4.216)	0.030^***^ (5.905)
*Size*	0.676^***^ (13.450)	0.700^***^ (12.514)	0.682^***^ (13.537)	0.720^***^ (12.847)
*Tangibility*	0.014 (0.036)	0.194 (0.458)	0.064 (0.165)	0.223 (0.521)
*Age*	−0.071 (−0.583)	−0.127 (−1.007)	−0.089 (−0.729)	−0.138 (−1.104)
*ROA*	1.567^**^ (1.826)	2.124^**^ (2.285)	1.595^*^ (1.860)	2.207^**^ (2.386)
*Lev*	0.236 (0.688)	0.515 (1.416)	0.017 (0.044)	0.304 (0.767)
*Inlc*	−0.506 (−0.515)	−1.665 (−1.180)	−0.601 (−1.180)	−1.613 (−1.142)
*Vrd*	0.008^**^ (2.657)	0.005 (1.517)	0.008^***^ (2.656)	0.005 (1.517)
*Constant*	−13.736^***^ (−6.271)	−13.796^***^ (−5.918)	−12.651^***^ (−5.840)	−13.235^***^ (−11.244)
LM	–	–	53.165 [0.000]	63.844 [0.000]
Wald F	–	–	77.539 {16.40}	83.818 {17.37}
*Year & Ind*	Yes	Yes	Yes	Yes
*N*	669	567	669	567
*Adj.R^2^*	0.326	0.358	0.328	0.369

*t* inspection value display. ***p < 0.01, **p < 0.05, and *p < 0.1.

The study conducts a further analysis according to the results of Models (1) and (2). On the whole, although senior managers’ psychological capital and employee equity incentives positively affect the quantity and quality of enterprise innovation output, it is clear that senior managers’ psychological capital (0.163 > 0.158) and employee equity incentives (0.217 > 0.105) have stronger positive effects on the quantity of enterprise innovation output. The launch of high-quality innovation activities has a higher failure rate (Ederer and Manso, 2013) and greater risks. For executives, on the one hand, there is a tendency to avoid risks due to the consideration of private rights and interests, so they are unwilling to carry out high-quality innovation. On the other hand, although some research shows that executives with high psychological capital may be more willing to innovate (Newman et al., 2014), there are many “obstacles” to enterprise innovation, including resource shortages and talent limitations. These constraints must be considered when executives make innovation decisions. For core employees, first of all, employee equity incentives link personal rights and interests with the innovation and development of the company. In this regard, the greater the value of innovation output, the more rights and interests employees obtain. Second, employee equity plans have a high tolerance for short-term enterprise innovation failure. The classified incentive effect generated by the employee equity plans incentivize employees to enhance their ability to bear innovation risks (Hall and Murphy, 2003). Thus, they become more inclined to high value, high-quality innovation. Finally, equity incentives encourage enterprises to create an environment conducive to innovation success, enabling core employees to share knowledge, resources, and technology with each other, and to collaborate as much as possible to improve the value of innovative products (Hochberg and Lindsey, 2010).

From the perspective of quantity and quality, the positive effect of employee equity incentives on the quantity of enterprise innovation output is greater (*Esop* = 0.217 > 0.158 = *Pyc*), while the positive effect of executive psychological capital on the quality of enterprise innovation output is clearly higher (*Esop* = 0.105 < 0.158 = *Pyc*). Senior executives are not only the decision-makers of innovation activities, but also the decision-makers of R&D capital investment, while core employees execute and participate in innovation activities. Enterprises must tolerate more failure, bear more risks, and invest more resources to improve the quality of innovation output, which depends on senior executives’ innovation decisions. Research shows that enterprise managers who possess higher psychological capital have better insight and creativity, and are more likely to implement breakthrough innovation with significant technological progress (Newman et al., 2014).

### 5.3. Endogenous treatment

According to the relevant theories and empirical findings mentioned above, whether to the quantity or quality of enterprise innovation, employee equity incentive plays a positive role. However, the integration of existing literature shows that both may have a cause and effect on each other. Companies that attach importance to innovation also tend to implement equity incentives to improve employees’ motivation to engage in innovation activities to produce more high-quality innovation results. This paper selects the appropriate instrumental variables, and then uses the two-stage least squares method to test the endogenous problems that may be caused by employee equity incentives and enterprise innovation. we reference existing practices (Hochberg and Lindsey, 2010; Chen et al., 2016), and select the natural logarithm of the number of employees [*Ln* (*Em*)] as the tool variable of this paper, The selected tool variables meet:

1. There is a correlation between the number of employees and the intensity of employee equity incentive.
2. The number of employees has nothing to do with the innovation output of the enterprise. These two points were verified in the first stage of regression.

The results of endogenous test are shown in Table 5 (showing the results of the model in the second stage, and Wald F statistics and LM statistics). The Wald F statistics in the first stage are significantly greater than the critical value under the 10% bias, indicating that the selected instrumental variables have a strong explanation for endogenous variables. The Kleibergen-Paap rk LM statistical results show that the value of *p* is 0.000, indicating that the problem of unrecognizability does not exist. In addition, the estimation results of the second stage are shown in Table 5, which are consistent with the benchmark regression results, indicating that the above research conclusions have strong robustness and reliability.

**Table 6:** Definition of alternative variables.

Variable properties	Variable names	Symbol	Variable calculation
Interpreted variables	Quantity of innovation outputs	*T_Innovation*	Ln(1 + The total number of patent authorizations lags behind by one phase)
	Quality of innovation output	*Q_Innovation*	Ln(1 + The number of invention patents granted lags behind one phase)
Explanatory variable	Staff equity incentive intensity	*Esop*	(Total number of shares granted to key employees/Total number of company shares)/Number of core staff incentives
Control variables	Enterprise growth	*Growth*	Growth rate of operating income: (Current year - Last year)/Last year
	Management shareholding ratio	*MngHold*	Number of shares held by management/Number of corporate equity

### 5.4. Robustness test

This paper tests the robustness of the empirical results by re-selecting alternative indicators for the explained variables, explanatory variables, and control variables as follows:

1. Replace the interpreted variables — Re-measure the quantity and quality of enterprise innovation output. The number of patent authorizations and patent applications can reflect the innovation output of enterprises. Compared with patent applications, patent authorizations certified by the National Patent Office can more accurately represent the effective innovation output of enterprises (Chang et al., 2015). However, their shortcomings are also obvious, and patent authorization often lags behind. Therefore, this paper uses the natural logarithm of the number of patent authorizations lagging behind one period as a substitute variable for the number of innovation outputs (*T_Innovation*). Similarly, it uses the natural logarithm of the number of patent authorizations lagging behind one period as a substitute variable for the quality of innovation outputs (*Q_Innovation*).
2. Replace explanatory variables — Re select the strength of employee equity incentive and the psychological capital of senior executives. In the usual regression analysis, the intensity of the employee equity incentives is judged by the proportion of equity incentives given to core employees from the company’s total equity. However, this method ignores the impact of per employee. Even if two companies with the same equity may have different incentive numbers, the *per capita* difference may be large. Therefore, this paper uses *per capita* employee equity incentive intensity as a substitute variable for employee equity incentive intensity (*Esop*).
3. Add control variables —Add two variables that affect enterprise innovation. Enterprises with high profitability and strong growth may invest more resources in R&D and innovation (Jugend et al., 2018). Therefore, this paper adds the growth rate of operating income as a new control variable that represents the growth of enterprises (*Growth*). Regardless of managers’ decisions, they will first weigh private interests. Enterprise innovation performance will change with the change of managers’ shareholding ratio (Morck et al., 1988). Therefore, this paper adds the management shareholding ratio (*MngHold*) as a new control variable (Table 6).

From Table 7, after the relevant indicators, such as explained variables, explanatory variables, and control variables are replaced, the relevant symbols in the robustness test results are consistent with the previous regression values, indicating that the research conclusions above are highly reliable.

**Table 7:** Robustness test results.

Variables	Replace interpreted variables		Replace explanatory variables		Replace control variables	
	*T_In*	*Q_In*	*T_In*	*Q_In*	*T_In*	*Q_In*
*Esop*	0.168^***^ (2.278)	0.154^***^ (2.278)	26.201^**^ (3.297)	13.073^*^ (2.126)	1.595^*^ (1.860)	0.706^***^ (12.651)
*Pyc*	0.064^*^ (1.573)	0.044^**^ (2.687)	0.104^*^ (2.034)	0.167^*^ (2.826)	0.022^***^ (4.216)	0.031^***^ (6.192)
*Constant*	−7.827^***^ (−7.171)	−10.516^***^ (−10.292)	1.004 (1.318)	−11.542^***^ (−9.619)	−10.372^***^ (−9.583)	−11.458^***^ (−9.632)
*N*	581	488	669	567	669	567
*Adj.R^2^*	0.298	0.365	0.343	0.360	0.328	0.355

*t* inspection value display. ***p < 0.01, **p < 0.05, and *p < 0.1.

### 5.5. Further study

#### 5.5.1. Verification of the intermediary effect of senior executives’ psychological capital on employee equity incentive and enterprise innovation

Table 8 mainly applies the recursive model constructed above to conduct a regression analysis on the intermediary effect of executive psychological capital on employee equity incentive and enterprise innovation. From the regression results of model 1 alone, the impact coefficient of employee equity incentives on the number of innovation outputs is *γ* = 0.139, passing the test at the 10% significance level. According to the regression coefficient of model 2 in Table 7, the impact coefficient of employee equity incentives on senior executives’ psychological capital is *α* = 0.279, passing the test at the 1% significance level. According to the empirical results of Model 3, the impact of employee equity incentives and senior executives’ psychological capital on the number of innovation output is statistically tested at a 10% significance level, with a regression coefficient of *γ*´ = 0.217, *δ* = 0.163. Regression results based on models 1–3 in Table 7, and according to the intermediary effect test process, senior managers’ psychological capital contributes to an intermediary effect between employee equity incentive and the number of enterprise innovations [refer to the calculation method of mediating effect by existing scholars (α·δ)]. The mediating effect of available executives’ psychological capital is about 0.045, accounting for 17.176% of the total effect.

**Table 8:** Verification of the intermediary effect of senior executives’ psychological capital.

Variables	*T_Innovation*			*Q_Innovation*		
	Model 1:*T_In*	Model 2:*Pyc*	Model 3:*T_In*	Model 4:Q*_In*	Model 5:*Pyc*	Model 6:Q*_In*
*Esop_m_*	0.139^*^ (3.693)	0.279^***^ (5.446)	0.217^**^ (4.726)	0.111^*^ (2.593)	0.264^***^ (4.587)	0.105^*^ (2.278)
*Pyc_m_*			0.163^*^ (2.564)			0.158^*^ (1.729)
*Constant*	−10.868^***^ (−10.671)	4.177^***^ (9.632)	−13.736^***^ (−6.271)	−12.221^***^ (−10.873)	4.275^***^ (8.202)	−13.796^***^ (−5.918)
*N*	669	669	669	567	567	567
*Adj.R^2^*	0.318	0.100	0.326	0.357	0.085	0.358
*Intermediary effect*	*α·δ =* 0.045			*α·δ* = 0.042		
*Proportion of effects*	*α·δ/*(*α·δ + γ´*) × 100 = 17.176%			*α·δ/*(*α·δ + γ´*) × 100 = 28.571		

*t* inspection value display. ***p < 0.01, **p < 0.05, and *p < 0.1.

Similarly, according to the empirical results of Model 4, the impact coefficient of employee equity incentives on innovation output quality is *γ* = 0.111, passing the test at the 10% significance level. Look at the regression value of model 5 alone, the impact coefficient of employee equity incentive on senior executives’ psychological capital is *α* = 0.264, passing the test at the 1% significance level. Analysis by regression value of model 6 alone, the impact of employee equity incentives and senior executives’ psychological capital on the quality of innovation output has passed the statistical test at the 10% significance level, and the regression coefficient si *γ*´ = 0.105, *δ* = 0.158. The regression values of models 4, 5 and 6 can be analyzed that senior executives’ psychological capital also has a partial mediating effect between employee equity incentives and enterprise innovation quality. The intermediary effect of executive psychological capital is about 0.042, accounting for 28.571% of the total effect. To sum up, the psychological capital of senior executives has a mediating effect between employee equity incentive and enterprise innovation. Therefore, Hypothesis H3a and H3b is verified.

According to the analysis of the results of Models 1–6, although the intermediary effect of executive psychological capital between employee equity incentive and innovation quality is small, it accounts for a slightly higher proportion of the total effect (28.571% > 17.176%). Risk bearing is an important driving force for enterprise innovation (Boubakri et al., 2013). High quality innovation activities tend to have longer cycles, are more affected by external uncertainties, and carry higher risk. As organizers of innovation activities and decision-makers of investment strategies, such as on human capital, executives have a risk aversion tendency for the sake of private interests. Some studies have pointed out that leaders with higher levels of psychological capital have stronger tenacity and motivation to implement breakthrough innovation (Newman et al., 2014; Russo and Stoykova, 2015), and they often achieve their goals by influencing employees’ innovation behavior. As an important incentive for enterprise innovation, equity incentives not only connect core employees, executivess and enterprises (Hochberg and Lindsey, 2010) and improves the risk-bearing level of core employees and executives but also helps stimulate executives’ willingness to carry out high-quality and high-risk innovation.

#### 5.5.2. The moderating effect of R&D investment on employee equity incentives, senior executives’ psychological capital, and enterprise innovation

Table 9 mainly applies the hierarchical adjustment regression model constructed above to conduct a regression analysis on the moderating effect of R&D investment on employee equity incentives and enterprise innovation. Based on the regression results of Models *T_*1 and *T_*2, it can be seen tha the cross projects of employee equity incentivel and R&D investment have a positive impact on the innovation output at the quantitative level, as well as the quality level (0.334,0.422), indicating that R&D investment positively regulates the connection between employee equity incentives and enterprise innovation. When combined with the four stages of the innovation process, the efforts of core employees determine the transformation effect of “R&D input into innovation output,” and sufficient R&D input can support employees in transforming more knowledge and technology into the desired innovation output (Kuo et al., 2017).

**Table 9:** The moderating effect of R&D investment on employee equity incentives and enterprise innovation.

Variables	*T_Innovation*		*Q_Innovation*	
	*T_*1	*T_*2	*Q_*3	*Q_*4
*Esop_e_*	0.217^**^ (4.726)	0.127^**^ (2.339)	0.105^*^ (2.278)	0.137^*^ (2.018)
*R&D*	0.208^***^ (4.074)	0.150^*^ (2.652)	0.032^***^ (6.332)	0.022^***^ (3.088)
*Esop_e_ × R&D*		0.361^***^ (3.102)		0.422^***^ (2.306)
*Constant*	−13.736^***^ (−6.271)	−10.352^***^ (−9.627)	−13.796^***^ (−5.918)	−11.537^***^ (−9.713)
*Year & Ind*	Yes	Yes	Yes	Yes
*N*	669	669	567	567
*Adj.R^2^*	0.326	0.338	0.358	0.367

*t* inspection value display. ***p < 0.01, **p < 0.05, and *p < 0.1.

Similarly, Table 10 shows the regression value of the test on the moderating effect of R&D investment between senior executives’ psychological capital and enterprise innovation. Based on the regression results of Models *Q_*3 and *Q_*4, the cross projects of senior executives’ psychological capital and R&D investment have a positive impact on the innovation output at the quantitative level, as well as the quality level (0.043 and 0.071), indicating that R&D investment positively regulates the connection between senior executives’ psychological capital and enterprise innovation. As a decision-making group of innovation activities, senior executives bear the responsibility of frustration and failure in innovation in response to innovation projects with long cycles, high risk, and high cost. At this time, senior executives’ psychological capital may become the key factor affecting innovation decisions. As a necessary resource for innovation, increased R&D investment undoubtedly enhances the innovation motivation and psychological capital of senior executives and encourages them to invest in innovative activities with high risks to maximize their returns and improve innovation performance.

**Table 10:** The moderating effect of R&D investment on executives’ psychological capital and enterprise innovation.

Variables	*T_Innovation*		*Q_Innovation*	
	*T_*1	*T_*2	*Q_*3	*Q_*4
*Pyc_e_*	0.163^*^ (2.564)	0.141^*^ (1.754)	0.158^*^ (1.729)	0.108^*^ (2.160)
*R&D*	0.208^***^ (4.074)	0.163^*^ (1.893)	0.032^***^ (6.332)	0.016^*^ (1.366)
*Pyc_e_ × R&D*		0.043^*^ (1.116)		0.071^*^ (1.489)
*Constant*	−13.736^***^ (−6.271)	−10.135^***^ (−9.012)	−13.796^***^ (−5.918)	4.275^***^ (8.202)
*Year & Ind*	Yes	Yes	Yes	Yes
*N*	669	669	567	567
*Adj.R^2^*	0.326	0.328	0.358	0.364

*t* inspection value display. ***p < 0.01, **p < 0.05, and *p < 0.1.

#### 5.5.3. Employee equity incentive forms, senior executives’ psychological capital, and enterprise innovation

This paper explores the positive effect of employee equity incentive and executive psychological capital on enterprise innovation, by regrouping and regressing the data according to the forms of equity incentives, based on whether there are differences due to different forms of employee equity incentive. Table 11 below shows the regression results. In the grouping of stock option form (O), reviewing the regression results of model, it can be seen that the impact coefficient of employee equity incentive on enterprise innovation output is 0.299 on the quantitative level and 0.253 on the quality level, respectively, passing the statistical test. Similarly, the regression coefficients of senior executives’ psychological capital are 0.153 and 0.057, respectively, also passing the statistical test, thereby indicating that the implementation of stock options can significantly promote enterprise innovation, and also enable the psychological capital of executives to maintain a positive role in enterprise innovation and development.

**Table 11:** Employee equity incentive forms, senior executives’ psychological capital, and enterprise innovation.

Variables	Stock option(O)		Restricted stock(R)	
	*T_Innovation*	*Q_Innovation*	*T_Innovation*	*Q_Innovation*
*Esop*	0.229^**^ (2.217)	0.253^*^ (2.574)	0.020^***^ (3.772)	0.280 (0.122)
*Pyc*	0.153^**^ (1.307)	0.057^*^ (1.760)	−0.163 (−1.626)	−0.204^**^ (−2.137)
*Constant*	−11.640^***^ (−6.641)	−13.491^***^ (−6.327)	−9.582^***^ (−7.511)	−10.336^***^ (−7.650)
*Year & Ind*	Yes	Yes	Yes	Yes
*N*	216	167	534	466
*Adj.R^2^*	0.411	0.438	0.298	0.336

*t* inspection value display. ***p < 0.01, **p < 0.05, and *p < 0.1.

In the restricted stock form (R) grouping, the comprehensive results of models show that the positive effect of employee equity incentives on innovation output is limited, and the regression coefficient is only 0.020. Employee equity incentive has nothing to do with the quality of innovation output, and the correlation coefficient has not passed the statistical test. In addition, executives’ psychological capital negatively affects the quality of innovation output, with a regression coefficient of 0.204, which passes the significance test. Regression results in the comprehensive table, when compared to the implementation of stock options, the implementation of restricted stocks not only reduces the incentive effect on enterprise innovation but also makes senior executives’ psychological capital negatively impact enterprise innovation. Thus, Hypothesis H4a and H4b is verified.

#### 5.5.4. The innovation effectiveness of employee equity incentive and executives’ psychological capital is affected by the nature of property rights

In order to judge whether the effectiveness of employee equity incentive and senior executives’ psychological capital have a positive effect on enterprise innovation and is affected by different property rights, this paper groups and regresses the sample data according to the property rights and also considers the characteristics of China’s relevant systems. Based on the above grouped regression results of state-owned enterprises (as shown in Table 12), it can be seen that employee equity incentive and senior managers’ psychological capital have little positive effect on the number of innovative outputs of enterprises, and their regression coefficients are 0.081 and 0.030, respectively, passing the test at the 5% significance level. At this time, although the impact of employee equity incentive and executive psychological capital on the quality of innovation output is positive, it has not passed the statistical test.

**Table 12:** The innovation effectiveness of employee equity incentive and executives’ psychological capital is affected by the nature of property rights.

Variables	State-owned enterprise		Non-state-owned enterprises	
	*T_Innovation*	*Q_Innovation*	*T_Innovation*	*Q_Innovation*
*Esop*	0.030^**^ (2.539)	8.689 (0.607)	0.179^**^ (2.817)	0.148^*^ (2.216)
*Pyc*	0.081^**^ (2.041)	0.092 (0.166)	0.122^**^ (2.247)	0.156^***^ (3.648)
*Constant*	−11.949^***^ (−3.385)	2.515 (0.568)	−10.693^***^ (−8.475)	−11.043^***^ (−8.541)
*Year & Ind*	Yes	Yes	Yes	Yes
*N*	62	35	607	531
*Adj.R^2^*	0.526	0.562	0.302	0.328

*t* inspection value display. ***p < 0.01, **p < 0.05, and *p < 0.1.

Based on the above results of grouping regression for non-state-owned enterprises, both employee equity incentives and senior executives’ psychological capital can effectively promote the number of innovative outputs of enterprises, with regression coefficients of 0.179 and 0.122, which pass the statistical test. Similarly, both pages can positively affect the quality of enterprise innovation output, with regression coefficients of 0.148 and 0.156, both of which pass the statistical test. To sum up, it is found that the nature of property rights affects the promotion of executives’ psychological capital and employee equity incentives on enterprise innovation. Comparing the regression results in the table, state-owned enterprises will reduce the positive effects of executives’ psychological capital and employee equity incentive. Thus, Hypothesis H5 is verified.

## 6. Conclusion and policy enlightenment

This paper takes listed companies that implemented equity incentives in the A-share market from 2010 to 2021 as research samples to empirically examines the difference between the impact of executive psychological capital and employee equity incentives on enterprise innovation, and discusses the intermediary effect of executive psychological capital between the latter two. The research shows that employee equity incentives and executives’ psychology can effectively promote the increase in the number of innovative outputs of enterprises and significantly bring about the enhancement of output quality. Moreover, it also finds that employee equity incentives has a more significant positive effect in terms of quantity, while the psychological capital of senior executives has a better positive effect on innovation in terms of quality. Further research shows that there is an intermediary effect between employee equity incentive and enterprise innovation. However, this intermediary effect has a more significant impact on innovation output at the quality level. In addition, R&D investment positively regulates the relationship between employee equity incentive, executive psychological capital, and enterprise innovation. This study also found that the nature of property rights and the form of employee equity incentive affect the effectiveness of the positive role of employee equity incentive and executives’ psychological capital. Moreover, the implementation of restricted stocks will reduce the positive role of both on enterprise innovation, while the positive effect of both in state-owned enterprises is not high.

This paper provides a new basis for enterprises to promote the reform of employee equity incentive mechanism, enhance senior executives’ psychological capital, and then achieve innovation-driven development from the theoretical and practical aspects. According to the previous research results, the following policy suggestions can be advanced.

Starting from employee equity incentive:

1. Employee equity incentive system can positively affect enterprise innovation, but still needs to improve the design of the government mechanism. For example, at this stage, there is a lack of legal systems that match the equity incentives and that can protect employees’ rights and interests from injury, which makes the incentive effect of employee stock ownership of enterprises insufficient. In addition, the government’s existing relevant laws and regulations focus more on interest binding.
2. In order to give full play to the innovation-oriented effectiveness of employee equity incentives, we should ensure the effectiveness and scientific of the incentive plan. For example, according to the above empirical evidence, the proportion of stock options should be increased in the form of employee equity incentive to maintain a greater effect of equity incentives.
3. The design of employee equity incentive policy system should not only consider the changes in China’s stock market environment, but also pay attention to whether the sustainable development of enterprise innovation is affected by the proportion of state-owned property rights.

From the perspective of senior executives’ psychological capital:

4. The psychological capital of senior executives can enhance the innovation willingness of the organization as a whole. Enterprises should hire or increase the cultivation of managers with strong psychological capital to provide impetus for the growth of long-term value of enterprises.
5. Enterprises actively pursuing innovation should build a corresponding mechanism environment to give play to the innovation effect of senior executives’ psychological capital; for example, optimize the incentive mechanism for senior executives, improve the tolerance for senior executives’ innovation failure, promote the creation of innovation remedy effect, and then reduce the problem of myopia, establish a cultivation mechanism that can enhance senior executives’ long-term strategic vision, and urge them to focus on the future value brought by high-quality innovation of enterprises.
6. The government should play the role of strategic guidance, encourage enterprises to actively explore innovative projects, and then provide an external environment for giving play to the enterprise managers’ adventurous spirits.

## Data availability statement

The raw data supporting the conclusions of this article will be made available by the authors, without undue reservation.

## Author contributions

LY contributed to the generation of research concepts and the arrangement of theoretical frameworks. JH wrote the paper after completing data collection and analysis. All authors contributed to the article and approved the submitted version.

## Funding

The completion of this paper is thanks to the support of the National Bureau of Statistics Preferred Project (2021LY087), Key Program of Natural Science Foundation of Zhejiang Province (Z21G030004) and the characteristic & preponderant discipline of key construction universities in Zhejiang province (Zhejiang Gongshang University-Statistics).

## Conflict of interest

The authors declare that the research was conducted in the absence of any commercial or financial relationships that could be construed as a potential conflict of interest.

## Publisher’s note

All claims expressed in this article are solely those of the authors and do not necessarily represent those of their affiliated organizations, or those of the publisher, the editors and the reviewers. Any product that may be evaluated in this article, or claim that may be made by its manufacturer, is not guaranteed or endorsed by the publisher.

## References

[ref1] Aghion P. Van Reenen J. Zingales L. (2013). Innovation and institutional ownership. Am. Econ. Rev. 103, 277–304. doi: 10.1257/aer.103.1.277

[ref2] Baron A.and Armstrong M. (2007). Human Capital Managemnt: Achieving Added Value Through People. London: Kogan page Ltd.

[ref3] Baron R. M. Kenny D. A. (1986). The moderator-mediator variable distinction in social psychological research: conceptual, strategic, and statistical considerations. J. Pers. Soc. Psychol. 51, 1173–1182. doi: 10.1037/0022-3514.51.6.1173, PMID: 3806354

[ref4] Bernstein S. (2015). Does going public affect innovation? J. Financ. 70, 1365–1403. doi: 10.1111/jofi.12275

[ref5] Boubakri N. Mansi S. A. Saffar W. (2013). Political institutions, connectedness, and corporate risk – taking. J. Int. Bus. Stud. 3, 195–215. doi: 10.1057/jibs.2013.2

[ref6] Bradley D. Kim I. Tian X. (2016). Do unions affect innovation? Manag. Sci. 63, 2251–2271.

[ref7] Brander J. A. Zhang W. (2017). Employee relations and innovation: an empirical analysis using patent data. Econ. Innov. New Technol. 26, 368–384. doi: 10.1080/10438599.2016.1202523

[ref8] Burns N. Minnick K. Starks L. T. (2013). CEO tournaments: a cross-country analysis of causes, cultural influences and conse-quences. J. Financial Quant. Anal. 52, 519–551.

[ref9] Call A. C. Campbell J. L. Dhaliwal D. S. Moon J. R. Jr. (2017). Employee quality and financial repoting outcomes. J. Account. Econ. 64, 123–149. doi: 10.1016/j.jacceco.2017.06.003

[ref10] Chang X. Fu K. Low A. Zhang W. (2015). Non-executive employee stock options and corporate innovation. J. Financ. Econ. 115, 168–188. doi: 10.1016/j.jfineco.2014.09.002

[ref11] Chen C. Chen Y. Hsu P. H. Podolski E. J. (2016). Be nice to your innovators: employee treatment and corporate Innovation performance. J. Corp. Finan. 39, 78–98. doi: 10.1016/j.jcorpfin.2016.06.001

[ref12] Cho C. Halford J. T. Hsu S. Ng L. (2016). Do managers matter for corporate innovation? J. Corp. Finan. 36, 206–229. doi: 10.1016/j.jcorpfin.2015.12.004

[ref13] Cornaggia J. Mao Y. Tian X. Wolfe B. (2015). Does banking competition affect innovation? J. Financ. Econ. 115, 189–209. doi: 10.1016/j.jfineco.2014.09.001

[ref14] Den Hartigh R. J. R. Hill Y. (2022). Conceptualizing and measuring psychological resilience: what can we learn from physics? New Ideas Psychol. 66:100934. doi: 10.1016/j.newideapsych.2022.100934

[ref15] Ding D. Z. Akhtar S. Ge G. L. (2009). Effects of inter-and intra-hierarchy wage dispersions on firm performance in Chinese enterprise. Int. J. Hum. Resour. Manag. 20, 2370–2381. doi: 10.1080/09585190903239716

[ref16] Ederer F. Manso G. (2013). Is pay for performance detrimental to innovation? Manag. Sci. 59, 1496–1513. doi: 10.1287/mnsc.1120.1683

[ref17] Edmans A. Fang V. W. Lewellen K. A. (2017). Equity vesting and investment. Rev. Financ. Stud. 30, 2229–2271. doi: 10.1093/rfs/hhx018

[ref18] Fang H. Nofsinger J. R. Quan J. (2015). The effect of employee stock option plans on operating performance in Chinese firms. J. Bank. Financ. 54, 141–159. doi: 10.1016/j.jbankfin.2015.01.010

[ref19] Fang V. W. Tian X. Tice S. (2014). Does stock liquidity enhance or impede firm innovation? J. Financ. 69, 2085–2125. doi: 10.1111/jofi.12187

[ref20] Fatoki O. (2018). The impact of entrepreneurial resilience on the success of small and medium enterprises in South Africa. Sustainability 32:2527. doi: 10.3390/su10072527

[ref21] Francis B. Hasan I. Wu Q. (2015). Professors in the boardroom and their impact on corporate governance and firm performance. Financial Mang. 3, 547–581. doi: 10.1111/fima.12069

[ref22] Gao Q. Y. Wu C. S. Wang L. C. Zhao X. Y. (2020). The entrepreneur's psychological capital, creative innovation behavior, and enterprise performance. Front. Psychol. 11:1651. doi: 10.3389/fpsyg.2020.01651 32793048 PMC7393239

[ref23] Griliches Z. (1990). Patent statistics as economic indicators: a survey. J. Econ. Lit. 28, 1661–1707.

[ref24] Hall B. J. Murphy K. J. (2003). The trouble with stock options. J. Econ. Perspect. 17, 49–70. doi: 10.1257/089533003769204353

[ref25] Hallak R. Assaker G. O’Connor P. Lee C. (2018). Firm performance in the upscale restaurant sector: the effects of resilience, creative self-efficacy, innovation and industry experience. J. Retail. Consum. Serv. 40, 229–240. doi: 10.1016/j.jretconser.2017.10.014

[ref26] Hambrick D. C. Mason P. A. (1984). Upper echelons: the organization as a reflection of its top managers. Acad. Manag. Rev. 9, 193–206. doi: 10.2307/258434

[ref27] Hattori R. A. Lapidus T. (2004). Collaboration, trust and innovative change. J. Chang. Manag. 4, 97–104. doi: 10.1080/14697010320001549197

[ref28] Hirshleifer D. Low A. Teoh S. H. (2012). Are overconfident CEOs better innovators? J. Financ. 67, 1457–1498. doi: 10.1111/j.1540-6261.2012.01753.x

[ref29] Hochberg Y. V. Lindsey L. (2010). Incentives, targeting, and firm performance: an analysis of non - executive stock options. Rev. Financ. Stud. 23, 4148–4186. doi: 10.1093/rfs/hhq093

[ref30] Huang J. Sheng M. (2013). Do top management background characteristics have information content? Manage. World 9, 144–153.

[ref31] Jefferson G. H. Bai H. M. Guan X. J. Yu X. Y. (2006). R&D performance in Chinese industry. Econ. Innov. New Technol. 15, 345–366. doi: 10.1080/10438590500512851

[ref32] Jugend D. Jabbour C. J. C. Scaliza J. A. A. Rocha R. S. Junior J. A. G. Latan H.. (2018). Relationships among open innovation, innovative performance, government support and firm size: comparing Brazilian firms embracing different levels of radicalismin innovation. Technovation 74, 54–65. doi: 10.1016/j.technovation.2018.02.004

[ref33] Kuo C. I. Wu C. H. Lin B. W. (2017). Gaining from scientific knowledge: the role of knowledge accumulation and knowledge combination. Acad. Manag. Annu. Meet. Proc. 1:15029. doi: 10.1111/radm.12322

[ref34] Lafuente E. Vaillant Y. Vendrell-Herrero F. Gomes E. (2019). Bouncing Back from failure: entrepreneurial resilience and the internationalisation of subsequent ventures created by serial entrepreneurs. Appl. Psychol. 68, 658–694. doi: 10.1111/apps.12175

[ref35] Li T. Y. Ling W. Yu Z. J. Dang X. (2020). Analysis of the influence of entrepreneur's psychological capital on employee's innovation behavior under leader-member exchange relationship. Front. Psychol. 11:1853. doi: 10.3389/fpsyg.2020.01853 32903662 PMC7438721

[ref36] Luthans F. Avollio J. Avey J. B. Norman S. M. (2007). Positive psychological capital: measurement and relationship with performance and satisfaction. Pers. Psychol. 60, 541–572. doi: 10.1111/j.1744-6570.2007.00083.x

[ref37] Manso G. (2011). Motivating innovation. J. Financ. 66, 1823–1860. doi: 10.1111/j.1540-6261.2011.01688.x

[ref38] Mao C. X. Weathers J. (2019). Employee treatment and firm innovation. J. Bus. Financ. Acc. 46, 977–1002. doi: 10.1111/jbfa.12393

[ref39] Mao C. X. Zhang C. (2018). Managerial risk-taking incentive and firm innovation: evidence from FAS 123R. J. Financ. Quant. Anal. 53, 867–898. doi: 10.1017/S002210901700120X

[ref40] Mehta R. Dahl D. W. Zhu R. J. (2017). Socialrecognition versus financial incentives? Exploring the effects of creativity-contingent external rewards on creative performance. J. Consum. Res. 44, 536–553. doi: 10.1093/jcr/ucx062

[ref41] Minnick K. Noga T. (2010). Do corporate governance characteristics influence tax management. J. Corp. Finan. 16, 703–718. doi: 10.1016/j.jcorpfin.2010.08.005

[ref42] Morck R. Shleifer A. Vishny R. W. (1988). Management ownership and market valuation: an empirical analysis. J. Financ. Econ. 20, 293–315. doi: 10.1016/0304-405X(88)90048-7

[ref43] Muller A. Whiteman G. (2015). Corporate philanthropic responses to emergent human needs: the role of organizational attention focus. J. Bus. Ethics 137, 1–16. doi: 10.1007/s10551-015-2556-x

[ref44] Newman A. Ucbasaran D. Zhu F. Hirst G. (2014). Psychological capital: a review and synthesis. J. Organ. Behav. 35, 120–138. doi: 10.1002/job.1916

[ref45] Oyer P. Schaefer S. (2005). Why do some firms give stock options to all employees? An empirical examination of alternative theories. J. Financ. Econ. 76, 99–133. doi: 10.1016/j.jfineco.2004.03.004

[ref46] Rego A. Cavazotte F. Cunha M. P. E. Valverde C. Meyer M. Giustiniano L. (2021). Gritty leaders promoting employees’ thriving at work. J. Manag. 47, 1155–1184. doi: 10.1177/0149206320904765

[ref47] Russo S. D. Stoykova P. (2015). Psychological capital intervention (PCI): a replication and extension. Hum. Resour. Dev. Q. 26, 329–347. doi: 10.1002/hrdq.21212

[ref48] Santoro G. Bertoldi B. Giachino C. Candelo E. (2020). Exploring the relationship between entrepreneurial resilience and success: the moderating role of stakeholders’ engagement. J. Bus. Res. 119, 142–150. doi: 10.1016/j.jbusres.2018.11.052

[ref49] Siegel P. A. Hambrick D. C. (2005). Pay disparities within top management groups: evidence of harmful effects on performance of high-technology firms. Organ. Sci. 16, 259–274. doi: 10.1287/orsc.1050.0128

[ref50] Sihvola S. Kvist T. Nurmeksela A. (2022). Nurse leaders’ resilience and their role in supporting nurses’ resilience during the COVID-19 pandemic: a scoping review. J. Nurs. Manag. 30, 1869–1880. doi: 10.1111/jonm.13640, PMID: 35434873 PMC9115204

[ref51] Torp S. Nielsen B. B. (2018). Psychological ownership and financial firm performance: the interplay of employee stock ownership and participative leadership. Aust. J. Manag. 43, 476–492. doi: 10.1177/0312896218755517

[ref52] Tsang A. Wang K. T. Liu S. Yu L. (2021). Integrating corporate social responsibility criteria into executive compensation and firm innovation: international evidence. J. Corp. Finan. 70:102070. doi: 10.1016/j.jcorpfin.2021.102070

[ref53] Van Knippenberg B. Van Knippenberg D. (2005). Leader self-sacrifice and leadership effectiveness: the moderating role of leader prototypicality. J. Appl. Psychol. 90, 25–37. doi: 10.1037/0021-9010.90.1.25 15641888

[ref54] Wang Y. F. Chen Y. Zhu Y. (2021). Promoting innovative behavior in employees: the mechanism of leader psychological capital. Front. Psychol. 11:598090. doi: 10.3389/fpsyg.2020.598090 33510678 PMC7835524

[ref55] Wu J. Tu R. (2007). CEO stock option pay and R&D spending: a behavioral agency explanation. J. Bus. Res. 60, 482–492. doi: 10.1016/j.jbusres.2006.12.006

[ref56] Zaltman G. Duncan R. Holbek J. (1973). Innovations and organizations. New York: John Wiley & Sons.

